# Chloroquine-resistant *Plasmodium vivax *malaria in Debre Zeit, Ethiopia

**DOI:** 10.1186/1475-2875-7-220

**Published:** 2008-10-29

**Authors:** Hiwot Teka, Beyene Petros, Lawrence Yamuah, Gezahegn Tesfaye, Ibrahim Elhassan, Simon Muchohi, Gilbert Kokwaro, Abraham Aseffa, Howard Engers

**Affiliations:** 1 Department of Biology, Addis Ababa University, Addis Ababa, Ethiopia; 2 Armauer Hansen Research Institute (AHRI), Addis Ababa, Ethiopia; 3 Federal Ministry of Health, Malaria and Other Vector borne Diseases Prevention and Control, Addis Ababa, Ethiopia; 4 Kenya Medical Research Institute (KEMRI)/Wellcome Trust Research Programme, Centre for Geographic Medicine Research (Coast), Kilifi, Kenya; 5 Department of Pharmaceutics and Pharmacy Practice, School of Pharmacy, University of Nairobi, Nairobi, Kenya; 6 Institute of endemic diseases, University of Khartoum, Sudan

## Abstract

**Background:**

*Plasmodium vivax *accounts for about 40% of all malaria infection in Ethiopia. Chloroquine (CQ) is the first line treatment for confirmed *P. vivax *malaria in the country. The first report of CQ treatment failure in *P. vivax *was from Debre Zeit, which suggested the presence of chloroquine resistance.

**Methods:**

An *in vivo *drug efficacy study was conducted in Debre Zeit from June to August 2006. Eighty-seven patients with microscopically confirmed *P. vivax *malaria, aged between 8 months and 52 years, were recruited and treated under supervision with CQ (25 mg/kg over three days). Clinical and parasitological parameters were assessed during the 28 day follow-up period. CQ and desethylchloroquine (DCQ) blood and serum concentrations were determined with high performance liquid chromatography (HPLC) in patients who showed recurrent parasitaemia.

**Results:**

Of the 87 patients recruited in the study, one was lost to follow-up and three were excluded due to *P. falciparum *infection during follow-up. A total of 83 (95%) of the study participants completed the follow-up. On enrolment, 39.8% had documented fever and 60.2% had a history of fever. The geometric mean parasite density of the patients was 7045 parasites/μl. Among these, four patients had recurrent parasitaemia on Day 28. The blood CQ plus DCQ concentrations of these four patients were all above the minimal effective concentration (> 100 ng/ml).

**Conclusion:**

Chloroquine-resistant *P. vivax *parasites are emerging in Debre Zeit, Ethiopia. A multi-centre national survey is needed to better understand the extent of *P. vivax *resistance to CQ in Ethiopia.

## Background

*Plasmodium vivax *malaria is the most geographically widespread and the second prevalent cause of malaria globally. Among 2.6 billion people at risk of malaria infection, annual estimates of *P. vivax *cases range from 130 to 435 million [1], 90% of these infections occur outside Africa [2]. Ethiopia has the highest proportion of *P. vivax *malaria on the continent, accounting for approximately 40% of all infection in the country [3].

Chloroquine (CQ) is the first line treatment for *P. vivax *malaria in most parts of the world. However, CQ resistance in *P. vivax *has compromised its use since the first reported cases from Papua New Guinea in 1989 [4]. Since then, cases of resistance have been reported from places such as Indonesia, island of Nias, in Papua and Irian Jaya in 1991 [5], Myanmar in 1993 and 1995 [6,7], India in 1995 [8], Borneo in 1996 [9], and in south America; Guyana in 1996 [10], and parts of the Amazon Brazil [11-13], Columbia in 2001 [14], Vietnam in 2002 [15], and Peru in 2003 [16]. Resistance has also been reported from two areas in Turkey in 2004 [17]. To date prevalence as high as 84% of CQ-resistant *P. vivax *has been reported from the north-eastern coast of Indonesian Papua in 2003 [18].

CQ has also been the first line drug in Ethiopia for uncomplicated malaria since 1950. Following the findings of CQ treatment failure as high as 65% for *P. falciparum *in studies conducted at 18 sites between 1997/98 [19], sulphadoxine/pyrimethamine (SP) was introduced as a first line drug treatment for uncomplicated *falciparum *malaria in 1999 [20]. SP was replaced by artemether/lumefantrine (AL) five years later after a study conducted in 2003 in 11 sites showed 35.9% treatment failure at day 7 and 71.7% on day 28. The efficacy of AL was reported to be 99.1% in 2004, when it replaced SP [21]. However, CQ continued to be in use as first line drug for vivax malaria.

The first report of *P. falciparun *and *P. vivax *CQ treatment failure in Debre Zeit, was in 1995, when only 4 of 29 (14%) *P. falciparum *patients cleared their asexual parasites by day 7 and 2% of 225 *P. vivax *patients who were followed for seven days failed to respond to CQ treatment [22]. Malaria is one of the most frequently diagnosed acute illnesses and a principal cause of morbidity affecting all age groups in Debre Zeit. Transmission occurs throughout the year with *P. vivax *being more prevalent (70%) than *P. falciparum *(30%). Malaria transmission is generally more intense following the main rainy season, which occurs from September to December. The aim of the present study was to determine the rate of CQ treatment failure in *P. vivax *and confirm whether treatment failure is due to drug resistance or sub-therapeutic antimalarial drug concentrations.

## Methods

### Study site

**Figure 1:**
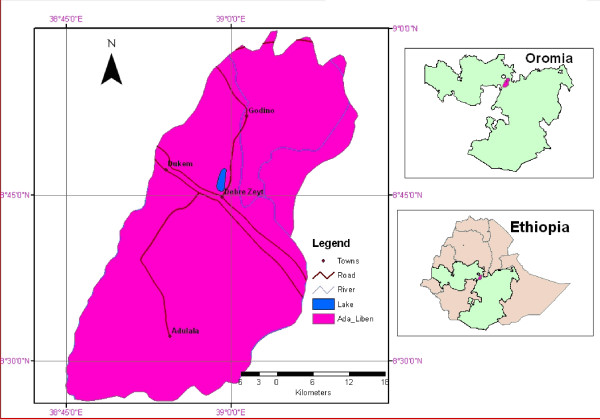
Map of the study site.

The study was conducted in Debre Zeit, the capital town of Adea Liben district, which covers an area of approximately 1,635 km^2^. It is located in Oromia region (Figure 1), Central Ethiopia and is about 45 km from Addis Ababa. An estimated population of 367,534 people live in this area [23]. Its altitude ranges from 1,500–2,200 m asl. It has a short rainy season from March to April and a long rainy season from June to September. The average annual rainfall recorded for the 10 years period (1996–2006) was 84.4 mm, while maximum and minimum temperatures over the same period were 28.6°C and 12.9°C, respectively [24].

There is one malaria centre where people with symptoms suggestive of malaria obtain free services for malaria diagnosis and treatment. Recruitment and follow-up for the present study was done at this centre.

### Sample size calculation

The sample size was calculated based on the expected proportion of *P. vivax *treatment failures with CQ in the study population. Assuming a maximum of 5% treatment failure in a population, a precision of 5%, and a 5% significance level, the formula, N = (Z/d)^2 ^P (1-P) was used for calculation. Assuming a loss-to-follow-up rate of 20% over a 28-day study, a total of 87 study participants were enrolled.

### Patient enrolment, follow-up and treatment

Patients were recruited according to the WHO protocol for monitoring anti-malarial drug resistance [25]. Patients aged between eight months and 52 years with symptoms suggestive of malaria illness, attending the malaria centre at Debre Zeit were screened for malaria infection using thick blood film. Those with *P. vivax *mono-infection with ≥250 parasites/μl of blood and elevated axillary temperature (≥37.5°C) or a history of fever within the previous 48 hours and who gave informed consent, or parent's or guardian's consent and assent (for children aged 12–17 years) were enrolled in the study. Patients were excluded if they had signs of severe malaria according to WHO criteria [26], had another obvious cause for their fever, had a history of allergy to CQ or lived outside the study area.

Following the treatment guidelines of the Federal Ministry of Health of Ethiopia (FMOH), 25 mg/kg of chloroquine phosphate (Batch Number 0073; Adigrat Pharmaceutical Factory, Addis Ababa, Ethiopia) was administered over a 3-day period (10 mg/kg on days 0 and 1 and 5 mg/kg on day 2) [27]. All drug doses were administered under the supervision of a member of the research team. The study participants were observed in case they vomited within 30 minutes of ingesting the drugs. Those who vomited the first dose were re-treated with a similar dose, while those who vomited twice were excluded from the study. Patients with axillary temperatures ≥ 38°C were treated with paracetamol (10 mg/kg). Patients who failed to respond to CQ treatment during the 28 days of follow-up were treated with oral quinine (8 mg/kg per day for seven days). The study participants were asked to return for follow-up evaluation on days 1, 2, 3, 7, 14, 21 and 28. Patients were encouraged to come back to the centre if they felt ill at any time during the follow-up period. Thick and thin blood films were prepared at times of all follow-up visits and additional blood samples were collected on filter paper at day 2 and any day after day 3 when patients had shown recurrent parasitaemia, for determination of serum CQ and DCQ concentrations. Patients who did not return to the malaria centre for follow-up assessment were traced to their homes.

Treatment success was defined as clearance of parasitaemia with no reappearance within the 28 days of follow-up period. Treatment failure due to CQ resistance was defined as parasitaemia recurred during the follow-up period in the presence of therapeutic concentrations of CQ and DCQ (i.e ≥ 100 ng/ml as determined using HPLC) [25,28].

The study was approved by the Addis Ababa University Ethical Review Committee, the AHRI/ALERT Ethical Review Committee and the National Ethical Review Committee.

### Laboratory analyses

#### Giemsa staining of thick and thin blood film and parasite density determination

Thick and thin blood film slides were prepared using 10% Giemsa solution for 30 min. The stained slides were examined under a light microscope using 100× oil immersion by an experienced laboratory technician. Parasitaemia was calculated per 200 white blood cells (WBC) assuming 8000 WBC/μl of blood [29]. A total of 300 fields were examined before a blood smear was considered negative. Ten percent of the slides were read by a second laboratory technician for quality control.

#### Drug quality assay

Drug disintegration, dissolution, identification and assay tests were done to assess the quality of the drug used in the study (chloroquine phosphate 250 mg containing 150 mg of chloroquine base, Batch Number 0073, Adigrat Pharmaceutical Factory, Addis Ababa, Ethiopia). The tested batch of CQ tablets was determined as per the British Pharmacopoeia (BP) [30].

#### Determination of CQ-DCQ concentrations on filter paper adsorbed blood

Blood concentrations of CQ and DCQ were measured using the HPLC method described by Bell *et al *[31], with some minor modifications for use in filter paper adsorbed blood samples. Quinidine (internal standard; 200 ng (20 μl of 10 ng/ml solution in methanol) was added to the blood sample spot (50 μl) adsorbed on Whatman filter paper (17 CHR) and allowed to dry at 37°C. The filter paper strips were cut into small pieces and put into labelled silanized pyrex glass PTFE-lined screw-cap tubes. Hydrochloric acid (2 M; 1 ml) was added, and the tubes vortexed for 10 seconds. Sodium hydroxide solution (5 M; 1 ml) was added, and the tubes vortexed for 10 seconds, followed by addition of the extracting solvent (tert-butyl methyl ether: n-hexane; 5 ml). The contents were mixed by mechanically inverting the tubes for 30 minutes using a tube rotor (Stuart STR4 rotator drive; Stuart Scientific, UK). The samples were then centrifuged (3000 rpm for 10 min at 4°C) to separate the phases. The upper organic phase was carefully transferred to a fresh silanized tube and evaporated to dryness at 37°C in a water bath under a gentle stream of white spot nitrogen gas (BOC Kenya Ltd, Nairobi). The extracted drug was reconstituted using mobile phase (100 μl) and an aliquot (50 μl) was injected onto the HPLC column. The mobile phase consisted of water and acetonitrile (90%: 10%; v/v) containing 1% (v/v) triethylamine, with pH adjusted to 2.8 with orthophosphoric acid.

The chromatographic system consisted of a gradient delivery pump (Spectra System P2000, Spectra Physics Analytical Inc. CA USA) connected to a syringe loading injector (Rheodyne Model 7125, CA USA) equipped with a 50-μl loop. Chromatographic separation was performed using a stainless steel analytical column (Synergi 4 μ Polar-RP 80Ä; 150 mm × 4.6 mm; Phenomenex^®^, Macclesfield, Cheshire, UK), preceded by a guard column (LiChroCart Lichrospher^® ^RP-18e; 10 mmx 4.6 mm; 5 μm; Darmstadt, Germany). A variable wavelength UV-visible detector (Spectra Series^® ^UV100; Spectra Physics Analytical Inc., CA, USA) set at 340 nm was used. Chromatographic data were recorded on a *ChromoJet *CH1 data integrator (Spectra Physics Thermo Separation Products, CA, USA).

### Statistical analysis

Data was double entered using Excel 2000 and was verified using Epi info 3.4 (Centre for Disease Control and Prevention, Atlanta, GA). STATA version 7.0 (Stat Corp., College station, TX, USA) was used for analysis. The survival analysis method of *in vivo *test was used. In this method patients who were excluded from the study due to *P. falciparum *infection or lost-to-follow-up during the course of the project were included in the analysis as non-treatment failure cases. Failure rate was derived from cumulative incidence of failure at day 28. Wilcoxon rank-sum tests were used in non-parametric comparisons, to determine the level of significant difference, p < 0.05 was taken as significant.

## Results

### Demographic and clinical characteristics of the study participants

A total of 2,422 consecutive patients self-reported to have symptoms of malaria, 87 patients who satisfied the inclusion/exclusion criteria were recruited. The median age of study participants was 16 years (range: 8 months to 52 years). Twenty point seven percent (n = 18) of the study participants were under five years of age. The mean ± standard deviation (SD) for duration of illness was 3.15 ± 1.85 days. Among the study participants, 62.1% had a history of fever and 37.9% had documented fever at the time of diagnosis. The geometric mean parasite density at day 0 was 6,614.6 parasites/μl (Table 1).

**Table 1:** Characteristics of patients enrolled in the *in vivo *efficacy test of chloroquine in *P. vivax *malaria from June-August 2006, Debre Zeit, Ethiopia.

**Characteristics**	
Total number of patients recruited	87

Number of patients under the age of five	20.7% (n = 18)

Age	
Median age	16 years
Range	8 Months – 52 Years

Sex	
Female	41.4% (n = 36)
Male	58.8% (n = 51)

History of fever	62.1% (n = 54)

Axillary temperature > 37.5°c (day 0)	37.9% (n = 33)

Duration of illness, Mean ± SD	3.15 ± 1.85 days

Geometric mean parasite density/μl (day 0)	6,614.6 parasites

SD = standard deviation

Symptoms such as fever, headache, chills/rigor, back pain, vomiting, myalgia, poor appetite, abdominal pain, cough, diarrhoea and joint pain were reported by the patients at the time of recruitment. Among these, the major ones were fever (96.5%), headache (86.2%) and chills/rigor (78%). Seven percent (n = 7) of the study participants vomited the first dose of CQ and they were retreated with a similar dose. None of them vomited a second time.

### Treatment response

Among the 87 study participants, three patients were excluded from the study due to *P. falciparum *infection during follow-up. One was found positive for asexual stages of *P. falciparum *on day 21 and the other two were positive for *P. falciparum *gametocytes on day 1 and 3. Only one participant was lost to follow-up while the remaining 83 study participants completed the 28-day follow-up.

Based on therapeutic failure risk calculated by the Kaplan-Meier survival analysis, the number of patients at risk on day 0 was 87 and these fell to 83 at day 28. Among the 83 patients, 42% (n = 35) had cleared their parasitaemia by day 1 and 98% (n = 81) by day 3 while 100% parasite clearance was observed by day 7. However, 4.6% (n = 4) of CQ treatment failure was observed during the follow-up period. This was observed in four children below seven years of age who developed symptomatic recurrent parasitaemia at day 28. This puts the risk of CQ failure on day 28 at 4.6% (Table 2).

**Table 2:** Estimates of risk of therapeutic failure of chloroquine in treatment of *P. vivax*, Debre Zeit, Ethiopia.

***D***	***N***	***TF***	***Ex***	***IRD***	***FCI*_*D*_**
Day 0	87	0	0	1.000	0
Day 1	87	0	1	1.000	0
Day 2	86	0	0	1.000	0
Day 3	86	0	1	1.000	0
Day 7	85	0	1	1.000	0
Day 14	84	0	0	1.000	0
Day 21	84	0	1	1.000	0
Day 28	83	4	0	0.954	0.046

Total		4	4		

*D *= day of test, *N *= number of subjects remaining at risk, *TF *= incident cases of therapeutic failure, *Ex *= excluded due to loss to follow-up, withdrawal or protocol violation, *IR*_*D *_= interval risk, *FCI*_*D *_= cumulative incidence of therapeutic failure

Though the day 0 parasite density of patients with recurrent parasitaemia was high, the difference failed to reach statistical significance (Wilcoxon rank sum test p = 0.061) observed from the parasitic density observed at day 0 in the patients who cleared their parasitaemia.

### Blood concentration of CQ plus DCQ

To monitor CQ absorption, CQ plus DCQ (CQ-DCQ) serum concentration of 83 samples (four patients with recurrent parasitaemia and 79 patients with treatment success) was determined on day 2. The median CQ-DCQ concentration was 882.3 ng/ml (range: 166.8–6714.6 ng/ml). There was no significant difference observed in the CQ-DCQ concentration at day 2 between the patients who showed recurrent parasitaemia and patients who had cleared their parasitaemia within the 28 days follow-up period (Wilcoxon rank-sum test, p = 0.52).

In order to confirm the cause of clinical treatment failure observed in the present study, blood drug concentration of CQ-DCQ was determined. The concentrations of CQ-DCQ in the blood at day 28 for patients who showed recurrent parasitaemia were all above the minimal effective concentration (MEC, 100 ng/ml) (Table 3). Furthermore, CQ and DCQ concentrations above MEC were also measured for samples of three patients who had shown adequate response to CQ treatment (Table 4).

**Table 3:** Chloroquine (CQ) plus desethylchloroquine (DCQ) concentration in the blood of *P. vivax *malaria patients with recurrent parasitaemia, Debre Zeit, Ethiopia, 2006.

		**Blood CQ+DCQ concentration (ng/ml)**	
		
**Case No**	**Age (years)**	**Day 2**	**Day 28**
02	2.4	829.9	524.6

37	7	486.1	672.1

57	3.4	1557.8	868.6

84	5	1611.9	1164.0

**Table 4:** Chloroquine (CQ) plus desethylchloroquine (DCQ) concentration in the blood of *P. vivax *malaria patients who have adequate clinical and parasitological response, Debre Zeit, 2006.

		**Blood CQ+DCQ concentration (ng/ml)**	
		
**Case No**	**Age (years)**	**Day 2**	**Day 28**
61	2.7	759.8	218.4

79	5	816.6	297.3

46	8	1,447.1	521.8

PCR was run on five paired samples (four from patients who had shown recurrent parasitaemia and one patient who had cleared parasitaemia during the follow-up period). However, it was only possible to amplify five isolates: four day 0 samples and one day 28 sample using primers published by Bruce *et al *[32]. Comparison of day 0 and day 28 samples was only possible for one patient showing the existence of two parasite populations in which one strain was not detected from samples that were collected at the time of recruitment.

## Discussion

According to the WHO protocol, resistance is defined as the presentation of signs of severe malaria within the first two days after supervised treatment or the presence of parasitaemia and axillary temperature > 37.5°C between day 3 and 28 or presence of parasitaemia on any day between day 7 and day 28, irrespective of clinical conditions [25]. In addition, evidence of CQ-DCQ concentrations above the minimal effective concentration (MEC) in the blood demonstrates the presence of resistance regardless of the origin of the parasite; whether it relapses from liver or is a recrudescence from blood stage [28,33]. In line with this definition, the present study provides evidence for the existence of CQ resistance in *P. vivax *in Debre Zeit, Ethiopia. This was confirmed with a quantitative analysis of the blood concentrations of CQ-DCQ, which were above MEC (100 ng/ml of blood). In addition, the presence of CQ-DCQ concentrations on day 2 (median = 882.3 ng/ml; range: 166.8–6714.6 ng/ml) showed that CQ was well absorbed from the gastrointestinal tract indicating that there were no other factors that might have influenced the kinetics of the drug including rapid excretion or poor absorption. Although only three samples from patients with successful treatment response were tested, blood concentrations of CQ-DCQ of these patients were above the MEC, which is in accord with a previous report by Baird *et al *[28].

This is the first report of CQ resistance in *P. vivax *from Africa. A previous report by Tulu *et al *in Debre Zeit has showed the presence of 2% (255) treatment failures in using seven days *in vivo *efficacy test [20]. However, serum drug concentrations were not determined in order to confirm drug resistance.

In addition to the determination of drug concentrations in patient blood samples, molecular genotyping is recommended in studies of anti-malarial drug efficacy [25]. This method is used to distinguish between relapse/recrudescence and re-infection in *P. vivax*. In present study, *msp 3-alpha *was used to genotype *P. vivax *isolates from patients who showed parasitaemia. The primers failed to amplify parasite isolates which could be due to the mismatch at the 3' end of the primers, thus not attached to the parasite DNA which, lead to an inconclusive result for the genotype. One limitation of this study is that it did not explore other marker genes, including *Pvcsp *and *Pvmsp1*, which had been applied elsewhere for genotyping in *P. vivax *parasite isolates to obtain additional information on the diversity of the resistant isolates [34].

Though genotyping is recommended in differentiating relapse/recrudescence and re-infection, it has shortcomings when used for *P. vivax *as this method assumes that the primary parasite population is the same as in recurrent parasitaemia due to relapse [35]. However, recent studies from three different countries have shown the presence of heterologous hypnozoites in the liver [36] in which a single allelic type of hypnozoite is being activated at a time [37], thus leading to misclassification of a recrudescence as a new infection. In addition, this assumption does not take into account the possibility of few strains circulating at low parasite density that cannot be detected in primary samples but could emerge to cause treatment failure, which again leads to misclassification of the treatment response [33]. Moreover, lack of a standardized classification of the outcome in categorizing mixed genotypes as recrudescence or re-infection is another limitation of this method. Therefore, in order to detect the emergence of drug resistance in *P. vivax*, clinical follow-up in combination with determination of CQ-DCQ concentrations in the blood at a time of recurrent parasitaemia will give a definitive diagnosis rather than follow-up in combination with genotyping. Since mixed parasite strains were observed in day 28 samples, when compared to day 0 sample, it was difficult to categorize as recrudescence/relapse from new infection.

In Ethiopia, there is a surveillance system for assessing the efficacy of first line drug for the treatment of *P. falciparum*. However, there have been no systematic studies for *P. vivax*. The present data confirmed the emergence CQ resistant *P. vivax *in the Debre Zeit. Even though the level of resistance is not so high as to require a treatment policy change, the finding will be useful in alerting the responsible authorities to monitor the level of drug resistance and to determine the extent of this problem in all parts of the country, bearing in mind that *P. vivax *malaria is responsible for 40% of malaria cases in Ethiopia.

## Conclusion

Chloroquine-resistant *P. vivax *parasites are emerging in Debre Zeit, Ethiopia. There is a need for regular monitoring of the pattern of resistance to antimalarial drugs in the country.

## Competing interests

The authors declare that they have no competing interests.

## Authors' contributions

HT was involved in all aspects of the project, data collection, analysis, interpretation, supervising the project at the study site, and determined CQ-DCQ concentrations using HPLC and in writing of the manuscript. BP, LY, HE, AA have made a contribution in the design, data interpretation, work supervision and in critically revising the manuscript. GT has made a contribution in identifying the problem and on the concept of the study. SM has assisted in conducting the HPLC method validation and determination of CQ-DCQ concentrations in the patients' blood samples, and helped in the analysis of the results. GK has facilitated the HPLC laboratory work at the Kenyan Medical Research Institute. IE has contributed ideas for the project and revised the manuscript. All authors read and approved the final version of the manuscript.
